# Down from the “Ivory Tower”? Not so much…Italian political scientists and the constitutional referendum campaign

**DOI:** 10.1057/s41304-021-00337-7

**Published:** 2021-06-10

**Authors:** Giulia Vicentini, Andrea Pritoni

**Affiliations:** 1 University of Siena, Via Mattioli 10, Siena, Italy; 2 University of Torino, Via Giuseppe Verdi, 8, 10124 Turin, TO Italy

**Keywords:** Constitutional referendum, Impact, Italy, Partisanship, Political scientists, Public visibility, Social relevance

## Abstract

Academics are often accused of being secluded in their “ivory towers”, focused on research and teaching but uninterested in, or unable to engage with, the public debate. If this is actually the case, under what conditions and at what particular moment is this likely to change? Following on three relevant dimensions—the visibility of political scientists, their partisanship and their impact in the public sphere—and combining press analysis with original survey data, this article has two main aims: first, to assess Italian political scientists’ (IPSs) social relevance in a period of huge political and institutional conflict such as the constitutional referendum held in December 2016; second, to explore the potential factors leading IPSs to be more or less present in the public debate. For the former, we focus on the public visibility of IPSs during the referendum campaign, as well as on the content of their public interventions, both concerning their neutral/partisan stance and their attitudes towards the constitutional reform. For the latter, we empirically test a few personal and institutional factors that are likely to influence individuals’ participation in the referendum debate.

## Introduction

Italy is a democratic polity where political science and the empirical social sciences in general are expected to have some impact on the public sphere, notwithstanding their difficult academic consolidation (Morlino 1992). However, the historical and cultural reasons behind the late development of the discipline have somewhat hindered its public visibility. Moreover, its lack of “eclecticism” and hyper-specialisation have represented obstacles that prevent many Italian political scientists (IPSs) from communicating their ideas as part of the public debate (Capano and Verzichelli 2016).

The subject of institutional reforms is a typical testing area for the effectiveness of IPSs’ public engagement and social relevance, in a country that has always been depicted as a “difficult democracy”, and where the issue of modernising the system of government has been a central theme for at least 40 years (Cotta and Verzichelli 2007). For this reason, the constitutional referendum held on 4 December 2016 represents a unique occasion to test the role of public intellectuals and academic experts in driving the public debate about the future of the Italian institutional setting. Indeed, the debate on the constitutional reform put forward by the Prime Minister, Matteo Renzi, in 2014—and the consequent political conflicts—split the country for months in a discussion that touched the fundamentals of the Italian democratic system. The motivations behind the debate reached far beyond the technical content of the reform: on the one hand, the Prime Minister personalised the debate, presenting the reform as a fundamental turning point for the country. On the other hand, some of his opponents even warned that the reform placed Italian democracy in danger (Vampa and Vignati 2017). Accordingly, the constitutional reform had a clear political value and might have represented a “critical juncture” for Italy (Pritoni et al. 2017) even if, in the end, it did not produce any institutional change because the proposals were rejected by the popular vote.

Political defeat in the constitutional referendum represented the end of Matteo Renzi’s time as Prime Minister and, in turn, gave rise to a series of political events that—in the following year and a half—led to the creation of the first “entirely populist” government in the history of the Italian republic (Bressanelli and Natali 2019). A period of such considerable political and institutional conflict may have opened up an opportunity for political scientists to “descend from their ivory tower” and engage in discussions with other social actors.

In this regard, the article aims to provide an explorative contribution based on the theoretical framework presented in this special issue (Real-Dato and Verzichelli 2021). To the best of our knowledge, there are no previous studies concerning the social relevance of public intellectuals during the Italian referendum campaign, nor a specific strand of literature dealing with the role of academics in public debate in Italy. We therefore rely on original data intended to address a very specific scope of research—namely, the role of IPSs within the referendum’s public debate. However, our analysis first assesses the visibility of IPSs during this political turning point and analyses whether their participation in the public debate actually reflected the expertise and bias of a few specialists against the indifference of many other scholars. Through original survey data, we also explore to what extent new attitudes towards partisanship, visibility and impact on public sphere emerged during such a critical juncture. We control these new attitudes by personal and academic characteristics and by various kinds of relations with the media. This way, we aim to identify the roles played by IPSs—observers, partisans, brokers, etc.—and assess the overall strength of their voices in terms of their impact on the public sphere. Finally, we are interested in exploring the determinants of IPSs’ participation in the public debate as a manifestation of their social relevance.

This article is organised as follows: after sections introducing the core questions and the methods chosen, the third section contextualises the institutional conflict at stake, summarising the content of the proposed constitutional reform and the public mobilisation around it. The following section presents the findings of survey data and press analysis in terms of IPS visibility in the media during the referendum campaign. We also employ survey data to explore the main drivers of IPS public engagement in the considered period. The final section systematises the empirical findings and presents a few concluding remarks on the visibility, partisanship and impact of IPSs during the referendum debate.

## Research questions and methods

We consider the Italian 2016 referendum as an interesting case study in which IPSs (or some of them) could have been mobilised and stimulated by the political conjuncture. It also offers comparative food for thought with respect to other articles included in this special issue, especially those on Greece and Spain (Zirganou-Kazolea and Tsirbas 2021; Real-Dato et al. 2021), both dealing with other highly salient domestic issues of political and institutional conflict developed around a controversial referendum. In fact, the constitutional reform proposed by the Renzi Government was an attempt to emerge from a long phase of institutional crisis that started in the 1980s, exploded in the 1990s, but remained unsolved by 20 years of political alternation.

Accordingly, this article has two main aims. First, we try to assess IPSs’ visibility, partisanship and impact during the constitutional referendum campaign. Second, we explore the potential factors leading IPSs to be more or less present in the public debate. Both aims derive from a unique (and fundamental) research question: did the constitutional referendum debate represent an opportunity for IPSs to “descend from their ivory tower” and to shape public opinion?

For our first aim, we focus on two main analytical dimensions: the degree of visibility of IPSs (and of IPS as a discipline) during the referendum campaign, especially compared with other related academic areas (i.e. constitutional law); and the roles played by political scientists during this debate, in particular with regard to the content of their public interventions (above all, their neutral/partisan stance and their attitudes towards the constitutional reform). Here we can expect that the high political saliency and polarisation of the constitutional referendum campaign represented a good opportunity for IPSs to increase their public visibility and that the divisive and politically charged nature of the referendum would make their interventions more partisan.

Given that the link between academics’ public engagement and their personal or institutional characteristics is rather neglected in the literature, we do not have strong expectations regarding the drivers of greater or lesser engagement in the public debate. Overall, our exploratory idea is that both participation in the referendum campaign and self-perception regarding such participation—in general terms—was higher in comparison with other electoral moments. More precisely, we can expect that such involvement depends on a number of individual and contextual factors. First, participation could depend on the inner motivations of individuals, such as norms (intrinsic motivation) or incentives (extrinsic motivation). In this respect, individuals more convinced of the social relevance of political science—and who would therefore perceive their participation as more important—are also more likely to participate in the public debate.

Second, participation may also depend on other individual characteristics, such as seniority, academic position and gender. Older people are more likely to have previous media experience and tend to have had more time to build a strong reputation than younger people, which is fundamental to participation in public debates. The same holds true for scholars with permanent positions: their public reputation is generally higher than that of non-tenure scholars. Besides, because of the well-known gender bias characterising both Italian political science and the Italian media system, we may also expect that males participate more than females (Sensales et al. 2016).

Third, we can also expect that participation in the referendum debate is affected by institutional factors. In their search for experts, mass media may privilege individuals belonging to more visible and prestigious institutions. In this respect, research teams located in richer and stronger academic institutions where there is a larger critical mass of researchers (Capano and Verzichelli 2016) are thus more likely to be reached by national media. These institutions are mostly located in the North of the country or in large cities, where the main media have their headquarters.

Concerning the methods, we first employ a press analysis to empirically test the extent to which IPSs’ public visibility increased during the referendum campaign, comparing the 2016 referendum period with other phases, and controlling whether IPSs intervened more often than constitutional lawyers.^1^ More specifically, we look at the frequency and content of press interventions—opinion articles, interviews, letters, and so on—that IPSs made in the most popular daily newspapers in Italy during the ninety days preceding the referendum and the thirty days following it.

The next move is completing the analysis of the centrality of political science with respect to both the public debate in general and the 2016 referendum campaign in particular. We employ the PROSEPS survey dataset (Vicentini et al. 2019) to provide a general picture of the attitudes of IPSs towards media presence and public visibility. For the referendum, just a few months after the closure of the PROSEPS survey, we submitted an ad hoc survey addressed to the same Italian sample of political scientists, namely all the scholars coded by the Italian university system as SPS/04 (“Political Science”) at the end of 2016.^2^ Following this, we merged the two datasets in order to analyse the findings both separately and in conjunction. This merged dataset allows us to produce an explorative of respondents’ participation in the traditional media debate during the referendum campaign.

## The 2016 constitutional referendum: an opportunity for IPSs to achieve media visibility (and, in turn, impact in the public sphere)?

On 4 December 2016, Italian voters were for the third time since 2001, and again in 2020—asked to vote on a constitutional reform approved by the Italian Parliament.^3^ Of these four reforms,^4^ this was considered to be the most important, because of its content, the marked politicisation of the referendum campaign, and the political and institutional consequences the vote would have (Pritoni et al. 2017). The proposed constitutional reform had five main components. First, it aimed to reduce the legislative powers of the Senate, which according to the proposition should only represent only regional institutions. Furthermore, the confidence vote to inaugurate or to dismiss the executive would have been limited solely to the lower house. The second main aspect dealt with the composition of the Senate, which, according to the proposal, would feature 100 indirectly elected senators rather than the current 315 directly elected senators. Third, the proposal included the abandonment of “symmetrical bicameralism”, which would have differentiated between two main legislative procedures: a more common unicameral procedure (with the Senate playing a merely consultative role) and a rarer bicameral procedure (in which a bill requires approval from both Chambers). The fourth main theme of the reform concerned the relationship between State and regional competences, drawing a different partition between matters reserved to the State and those devolved to the regions. The so-called concurrent competence, according to which State law legislates the principles that are later to be implemented by regional laws, was to be abolished, and all concurrent matters reassigned to the competence of either the State or the regions. Finally, the proposal abolished the CNEL (National Council for Economics and Labour) and removed the Provinces (the second-level administrative layer) from the Constitution.

The breadth and importance of the proposed changes implied a significant political battle and a considerable public debate. All the parties supporting the government led by Matteo Renzi (Democratic Party, New Centre-Right, Liberal Popular Alliance and Civic Choice) were united in advocating for the reform, while all opposition forces, from both the centre-right and the left, campaigned against it. In this regard, the opponents of the reform were extremely heterogeneous. Indeed, some prominent leading figures of the left-wing minority within the Democratic Party also lined up against the reform (Pritoni and Valbruzzi 2017).

On the one side, proponents argued that the changes would limit traditional Italian political dysfunction, such as (1) the excessive fragmentation of the party system (Sartori 1976; Cotta and Verzichelli 2007); (2) a byzantine legislative process (Di Palma 1977) with an abundance of “*leggine*” and a few “big reforms” (Kreppel 1997; Pritoni 2017); (3) very short-lived governments (Curini 2011) unable to fulfil their electoral pledges (Moury 2011). On the other side, opponents expressed four main concerns: (1) the way in which the reform was approved, without broad consensus; (2) the weakness of the new Senate, with very limited legislative powers and representation for the territories; (3) the high risk of legislative conflicts between the two chambers; (4) the reduced autonomy of the regions. In sum, they feared an excessive centralisation of powers around the government and, in turn, the possibility of an “autocratic drift” (Pasquino 2017). All these things considered, the political and institutional conflict arising from the reform was likely to provide fertile ground for the public engagement of experts in the field, such as political scientists.

## Visibility and roles of IPSs during the constitutional referendum campaign

In general, IPSs appear to be quite sceptical with regard to their visibility and impact in the public debate compared to their colleagues in other countries: the PROSEPS survey^5^ shows that a significant group of Italian respondents (64%) are convinced that “political science research very rarely makes it into the public debate”. Moreover, the proportion of IPSs who think they have a considerable impact on the general public is 16 percentage points below the European average (Table 1).

**Table 1:** Public visibility, social impact, media participation and normative patterns. *Source*: PROSEPS Survey—Cost Action “Professionalisation and Social Impact of European Political Science”

	Italy %	Europe %
*Overall, how do you evaluate the visibility in public debates/discussions of the research produced by political scientists in your country?*		
Not visible at all. No political science research ever makes it into the public debate	4.0	2.0
Scarcely visible. Very rarely does some PS research make it into the public debate	64.4	41.8
Quite visible. Occasionally, some PS research makes it into the public debate	29.9	42.8
Very visible. Very frequently PS research makes it into the public debate	1.1	12.2
I can't say/missing	0.6	1.2
*Regarding the impact of political scientists in comparison with other academics or public intellectuals, would you say that in your country:*		
Political scientists have no impact at all	13.0	5.1
Political scientists have a little impact on the general public	81.9	72.5
Political scientists have a considerable impact on the general public	3.4	19.5
I can't say/missing	1.7	2.9
*In the last 3 years, did you take part in public debates in the following media?*		
TV programs	22.6	33.2
Radio programs	29.9	41.4
Newspaper/magazines (including online outlets)	49.2	50.3
*To what extent do you agree with the following statements? (fully/somewhat agree)*		
PSs should engage in public debate since this is part of their role as social scientists	90.4	92.5
Political scientists should engage in public debate to expand their career options	17.0	41.0
PSs should engage in public debate in the media only after testing their ideas in academic outlets	59.9	57.4
Political scientists have a professional obligation to engage in public debate	43.5	70.1

Rather surprisingly, such a negative self-perception in terms of visibility and impact is not associated with an absence from the media: 59% of IPSs declare they took part in public debates in the media during the last 3 years, in line with the European average. However, the frequency of their interventions is particularly low, with only 20% of IPSs qualifying as “media activists”, namely respondents who intervened in at least two of the three traditional mass media over the last 3 years and did so very frequently or somewhat frequently (at least once every 3 months). Yet, IPSs do not lack “normative” motivation for public engagement, as around 90% of Italian respondents claim that political scientists should engage in public debate since this is part of their role as social scientists. However, the majority of them do not feel public engagement is a professional obligation and 83% do not even see any career advantage in it. However, the constitutional referendum could have represented a good opportunity for IPSs to increase their actual participation and visibility in the public sphere. We test this expectation in the rest of this section.

### *Results from the analysis of major newspapers*

Our first empirical exercise is to measure the media activism and visibility of IPSs during the referendum campaign. First, we examine the presence of IPSs in three popular daily newspapers in Italy (*Corriere della Sera*, *la Repubblica* and *la Stampa*) before and after the referendum (namely, from 4 September 2016 until 4 January 2017). We chose newspapers instead of other media because IPSs intervene more frequently in this type of outlet according to the PROSEPS survey. More precisely, we carried out a basic keyword search on the online archives of each newspaper: we looked at how many times the words “political scientist(s)” (*politologo/a/i/he*) and “constitutional lawyer(s)” (*costituzionalista/i/e*)^6^ occurred in the same 4-month period (including the referendum campaign period) over the last 5 years. Accordingly, the count may include any kind of direct interventions as well as indirect citations for both Italian and foreign political scientists, and the interventions may concern any kind of topic, not necessarily the constitutional reform.

**Fig. 1:**
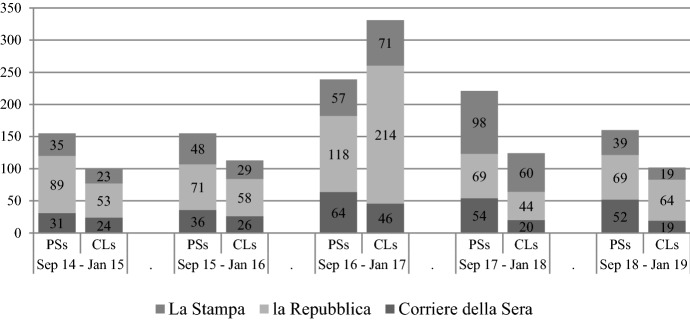
Press visibility of political scientists (PSs) and constitutional lawyers (CLs). *Source*: Authors’ own elaboration

Figure 1 seems to confirm that the press visibility of political scientists increased during the constitutional referendum campaign, but, proportionally, it increased much less than the visibility of constitutional lawyers, who are usually less visible. Yet, this very preliminary analysis does not clarify whether the increased visibility of political scientists during the last phase of 2016 was actually connected to their massive engagement in the referendum debate. To answer this question, we ran a more specific search based on the combination of the word “constitutional reform/referendum” (*riforma/referendum costituzionale*) and the name + surname of all the people with official affiliation in the ministerial disciplinary sector SPS/04 “Political Science” (including all full and associate professors and researchers with both permanent and non-permanent contracts) at the end of 2016 (219 people). In this case, and in contrast to the previous search, we decided to add a fourth daily newspaper: *il Sole 24 ore*. Notwithstanding the quite different nature of this economic newspaper in comparison with the other three “generalist” newspapers, we decided to broaden our analysis because *il Sole*’s attention towards political science is far from marginal, as it hosts some prominent IPSs among its columnists.^7^

The analysis suggests that, regardless of the relatively high number of occurrences of the terms *politologo* in the newspapers under consideration from 4 September 2016 to 4 January 2017, the number of “real” IPSs’ interventions or citations dealing with the constitutional reform is much smaller(63) and these refer only to 14 people, mostly well-known figures in the Italian public sphere.^8^ Therefore, this confirms the idea that media outlets tend to resort to the same set of individuals every time they need the opinion of a political scientist. In fact, the names that recur more often (18 and 11 times) coincide with those of the regular columnists of *il Sole 24 ore* and *la Repubblica,*, respectively. Thus it is quite likely they would have called upon anyway to intervene on various issues. We also analysed the content of the interventions of these political scientists. In this respect, it is worth noting that most interventions/citations (38 out of 63) were not neutral but partisan,^9^ with supportive interventions greatly outnumbering those opposing the reform (26 vs. 12).

Of course, the fact that only 14 IPSs out of 219 were cited in the newspapers we considered, does not mean that no other IPSs participated in the public debate during the referendum campaign. For instance, we found 20 IPSs who signed public appeals in favour of the reform, but only a couple of them were cited or called on to intervene in the four newspapers we considered. However, IPSs may have intervened in other national or local newspapers and/or via other media outlets such as radio and TV, and this is precisely what we have tried to detect with our ad hoc survey.

### *Results of the referendum survey*

The specific survey of IPSs’ participation and attitudes towards the 2016 referendum includes 97 fully completed questionnaires (response rate: 45%). The sample is a good approximation of the population of IPSs in terms of gender, academic position, geographical area and dimension of the higher education institution.^10^

With respect to the *levels of participation*, the findings confirm the limited public engagement of IPSs in the referendum campaign, already pointed out by the press analysis. In fact, according to survey responses, only a very small share of IPSs took part in the media debate on the constitutional reform (24 over 97: see Table 2, Q1) and, among them, more than a third did so only once (Q2). This very limited media presence during the referendum campaign seems to be consistent with IPSs’ perception. In fact, around 70% of respondents declared that IPSs were not particularly involved in the referendum public debate compared with other electoral moments, and 92% even stated that IPS involvement was low, especially if compared with other academic figures such as constitutional lawyers (Q4a-b).

**Table 2:** IPSs and the constitutional referendum: general overview. *Source*: Authors’ own data

Question	Potential answers	*N*	%
Q1. I took part in the public debate on the traditional media during the constitutional referendum campaign	Yes	24	24.7
	No	73	75.3
Q2. If so, with what frequency?	Around once a week	6	25.0
	Around once a month	9	37.5
	Only once	9	37.5
Q4a. PSs have not been particularly involved in the public debate compared to other electoral moments (general and European elections etc.)	Strongly agree (1–3)	36	37.1
	Somewhat agree (4–5)	32	33.0
	Somewhat disagree (6–7)	25	25.8
	Strongly disagree (8–10)	4	4.1
Q4b. PSs have not been particularly involved in the public debate compared to other academic figures (e.g. constitutional lawyers)	Strongly agree (1–3)	61	62.9
	Somewhat agree (4–5)	28	28.9
	Somewhat disagree (6–7)	4	4.1
	Strongly disagree (8–10)	4	4.1
Q4c. The majority of PSs who took part to the debate on the media just limited to scientifically analyse the pros and cons of the reform avoiding to publicly take a position	Strongly agree (1–3)	6	6.2
	Somewhat agree (4–5)	19	19.6
	Somewhat disagree (6–7)	33	34.0
	Strongly disagree (8–10)	39	40.2
Q4d. The majority of PSs supported the constitutional reform	Strongly agree (1–3)	22	22.7
	Somewhat agree (4–5)	57	58.8
	Somewhat disagree (6–7)	15	15.5
	Strongly disagree (8–10)	3	3.1
Q5. How did you vote at the 2016 constitutional referendum?	Yes	52	53.6
	No	26	26.8
	Prefer not to disclosure	19	19.6

Q4a/b/c/d represent the left end pole of a 10-point continuum where the opposite item is placed at the right end (semantic differential scale). We asked the respondents to place themselves on the 10-point scale

Our survey also allowed us to analyse the roles played by IPSs in the debate. Some questions explored general perceptions about the interventions of other colleagues in such a debate. According to more than 70% of the respondents, IPSs’ media interventions were not “neutral” but mostly “partisan” (Q4c); this is consistent with the findings we obtained from our press analysis.

We also asked IPSs about their perception of the general positioning of the category on the constitutional reform (Q4d), and the vast majority of respondents (81%) declared that, in their opinion, the majority of their colleagues were in favour of the reform. This is also consistent with the preliminary results we obtained from the press analysis. Q5 shows that, after all, the respondents’ “perception” of IPSs’ positioning on the reform was aligned with the electoral choice they actually made on 4 December 2016. In fact, according to our survey, an absolute majority of IPSs (54%) supported the constitutional reform at the ballot box, against 27% who voted “no”, and 20% who preferred not to answer this question.

In conclusion, on the one hand IPSs’ perceptions concerning their category orientation were quite consistent with the reality, suggesting a good level of “exchange” of opinions among colleagues. On the other hand, we do not observe any convergence between IPSs’ preferences and the general electorate, who largely rejected the reform (by a 59% majority).

However, another important aspect to investigate concerns the content of IPSs’ media interventions regarding the constitutional referendum campaign. In our survey we planned a series of questions focusing on the positioning, the type of approach and the content/style of those same interventions. As noted above, the number of respondents who declared that they had taken part in the referendum debate on the media was very small (24 people). Accordingly, the findings presented below need to be interpreted with caution given the low absolute numbers (Table 3).

**Table 3:** IPSs and the constitutional referendum: content of their media interventions. *Source*: Authors’ own data

Question	Potential answers	*N*	%
Q3a. I took a diagnostic approach: I explained the content of the reform and analysed (or supported/criticised) the positions of the actors involved	Strongly agree (1–3)	12	50.0
	Somewhat agree (4–5)	6	25.0
	Somewhat disagree (6–7)	4	16.7
	Strongly disagree (8–10)	2	8.3
Q3b. I used an academic/specialist style: I explained/supported/criticised the content and effects of the reform by using a specialist language and sometimes also referring to theories, authors, articles and books	Strongly agree (1–3)	8	33.3
	Somewhat agree (4–5)	8	33.3
	Somewhat disagree (6–7)	3	12.5
	Strongly disagree (8–10)	5	20.8
Q3c. I maintained a neutral position: I merely explained the content and effects of the reform without taking a position for or against it	Strongly agree (1–3)	8	33.3
	Somewhat agree (4–5)	2	8.3
	Somewhat disagree (6–7)	6	25.0
	Strongly disagree (8–10)	8	33.3

The questions represent the left-end pole of a 10-point continuum where the opposite item is placed at the right end (semantic differential scale). We asked the respondents to place themselves on the 10-point scale

First of all, the survey confirms a more widespread diagnostic approach compared to a prognostic one: 75% of the IPSs participating in the public debate limited their interventions to explaining the content of the reform and to analysing the positions of the political actors involved, rather than acting as policy advisors suggesting alternative solutions or possible ameliorative elements. This is not particularly surprising, though, as they were mostly called on to comment on a reform that was already defined, and the referendum was not about selecting from various options but, rather, simply supporting or rejecting the proposed reform as a whole.

Secondly, only eight IPSs abandoned their professional/academic “angle”—which does not only mean using a specialised vocabulary, but also includes reference to theories, authors, articles and books in addressing the reform—in favour of a more “journalistic” approach, simplifying their language and content. On the contrary, two-thirds of our respondents (16 people) confirm that they used an academic style when intervening in the public debate. This is quite surprising, given the well-known tendency to simplification when called on to participate in a public debate, in order to reach as many citizens as possible. However, on this we also have to signal an element of caution: each IPS might have a different personal interpretation of what “specialised language” means in a media context.

Finally, and most importantly, we confirm that partisan interventions are more frequent than neutral ones: almost 60% of IPSs who intervened in the media publicly expressed a clear preference for either “yes” or “no”, explaining the pros and cons that would have arisen through the approval of the reform. In contrast, only ten IPSs opted for neutral interventions. However, this does not mean that IPSs disregarded the importance of neutrality. Simply, the “object” of the intervention is likely to affect the nature of the intervention itself: while it would be disappointing to hear political scientists publicly expressing their own party preferences on the eve of an election, it would be much harder to maintain a “neutral” stance when facing issues for which the electors have to express a “yes” or “no” vote. Furthermore, this predominance of “partisan interventions”—more often than not advocating “yes”—means that IPSs—or, at least, the few who actually participated in the referendum debate—tried to shape public opinion and, in so doing, to gain social relevance. However, precisely the fact that Italian citizens largely rejected the proposed reform is a preliminary confirmation of their (very) limited capacity to do so.

## Exploring the factors of IPSs’ public engagement during the constitutional referendum campaign

Our survey data allow us to assess whether IPSs’ participation in the referendum debate varied according to socio-demographic and academic characteristics, their relation with the media and various normative beliefs about the discipline. We can explore these potential relationships and inquire into the factors explaining participation in the debate by merging the PROSEPS and the referendum survey datasets presented above. As the two populations were slightly different and some people responded to one survey but not the other, we inevitably have a number of missing cases in the merged dataset. However, 70 people answered both surveys, which means a multivariate regression, including a representative sample of the IPS population, is still possible.

Table 4 presents the results of the statistical models testing a series of factors that could affect participation in the public debate in the traditional media during the referendum campaign (Q1 in Table 2) as the dependent variable. Given that this dependent variable is coded as either “0” or “1” on the basis of whether the respondent took part in the public debate, we present logistic regressions instead of the “traditional” ordinary least squares (OLS) model.

**Table 4:** Multivariate logistic regressions

Variable	Model 1 Institutional variables	Model 2 Individual variables	Model 3 Survey variables
Constant	− 0.491 (0.532)	− 1.888 (79.041)	− 20.488 (95.709)
*Institutional variables*			
Northern University	Ref. category	Ref. category	Ref. category
Central and Southern University	0.411 (0.576)	0.414 (0.586)	0.104 (0.674)
Mega and Big HEIs	Ref. category	Ref. category	Ref. category
Other HEIs	0.493 (0.631)	0.497 (0.636)	0.939 (0.767)
*Individual variables*			
Year of birth	–	0.001 (0.040)	0.010 (0.048)
Gender: male	–	Ref. category	Ref. category
Gender: female	–	− 0.134 (0.630)	0.456 (0.788)
Permanent contract	–	Ref. category	Ref. category
Temporary contract	–	0.207 (1.320)	− 0.377 (1.686)
*PROSEPS survey variables*			
Motivation	–	–	− 0.097 (0.349)
Political Science social relevance	–	–	− 0.349 (0.413)
Media activism	**–**	**–**	1.070*** (0.321)
*Diagnostics*			
N	69	69	69
Cox & Snell *R*^2^	0.022	0.023	0.197
Nagelkerke *R*^2^	0.032	0.034	0.290

Standard errors in parentheses; *p < 0.10; **p < 0.05; ***p < 0.01

As previously noted, in order to explore IPSs’ participation in the referendum public debate, we differentiate independent variables into three categories: (a) institutional variables; (b) individual variables; (c) PROSEPS Survey variables. In Model 1, we test the predictive power of institutional variables only; in Model 2, we add individual variables; in Model 3, we take into account all independent variables. In the first set of variables we present two dummies accounting for (1) the geographical zone (with Northern Universities as the reference category) and (2) the dimension (with Mega and Big Universities as the reference category) of the respondent’s higher education institution (HEI). In the second set of variables we include (1) the year of birth of the respondent (scale variable), (2) her/his academic position (dummy, with professors and researchers with a permanent academic position as the reference category) and (3) her/his gender (dummy, with males as the reference category). In the third set of variables we also have (1) a variable accounting for different levels of motivation to engage in the public debate, on the basis of the questions presented in Table 1,^11^ (2) a variable measuring the individual perception of relevance of Political Science in the public debate^12^ and (3) a variable measuring media activism.^13^ All PROSEPS Survey variables should influence the dependent variable directly: first, “normative” and/or “instrumental” motivations could be very important to explain the participation in the public debate; second, individuals considering Political Science as particularly relevant in the public debate could be more motivated to participate in the referendum campaign; third, previous experience in media participation could also foster participation in the referendum debate.

The empirical findings in Table 4 do not support the expectation that “institutional factors” matter for participation in the referendum campaign. IPSs in Northern universities did not participate any more than their colleagues from the rest of Italy; the same holds true for the size of the HEI. Neither do individual characteristics seem to matter so much: there is no statistically significant difference between males and females, nor with regard to academic position or age.

Among the variables originating from the PROSEPS Survey, the only factor that appears to be (very) relevant for participation in the referendum campaign is media activism: this means that the constitutional referendum did not represent an opportunity for *more* IPSs to be at the core of the public debate. In other words, those who participated in the public debate were *already* highly visible in *other* public debates. This suggests that IPSs who participated in the constitutional referendum campaign were people who frequently intervene in the media regardless of the (political) matter at hand. In these terms, while we might have expected that the greater politicisation of the referendum issue opened the door for more participants, this was not the case. Against this expectation, our findings suggest that IPSs’ participation in the public debate is highly structured.

## Discussion of the findings and concluding remarks

The aim of this article was to analyse the public role played by IPSs during a specific moment of institutional crisis represented by the constitutional referendum campaign at the end of 2016. We can try now to answer our main research question: did the constitutional referendum debate represent an opportunity for (more) IPSs to “descend from their ivory tower” and to shape public opinion? Unfortunately for the discipline, empirical findings seem to suggest that the answer is “not so much”.

That said, we try here to embed our findings in the general framework based on the three relevant dimensions (i.e. visibility, partisanship and impact). First, press visibility of political scientists apparently increased in the 4-month period before and after the referendum, but the link between this quantitative increase and the referendum debate is not confirmed by the following press-analysis, intended to identify all IPSs’ interventions concerning the constitutional reform. Not by chance, proportionally, IPSs’ visibility has increased by less than that of constitutional lawyers. Second, IPSs’ interventions in the four major newspapers in Italy concerning the constitutional reform were restricted to only a very few persons, mostly regular columnists of the newspapers considered. Third, IPSs perceived their own professional community as being scarcely involved in the constitutional referendum debate. This does not seem to change very much depending on socio-demographic, academic characteristics, particular beliefs concerning the social relevance of political science or normative motivations to engage in the public debate. Rather, the referendum campaign saw the participation of a few “usual suspects” *mediatic scholars*—namely those political scientists, we regularly see in the public debate, whatever the issue—*vis-à-vis* a large majority of *invisible* ones. This means that the referendum did not represent an opportunity for more people to intervene in the media and (try to) shape public opinion on that topic, in contrast to constitutional lawyers. In this regard, the presence of a mediatic minority *vs* an invisible majority of IPSs resembles the situation of most of the cases analysed in this special issue, especially those of Greece, Israel and (to a lesser extent) Spain (Zirganou-Kazolea and Tsirbas 2021; Neubauer-Shani 2021; Real-Dato et al. 2021).

In this regard, the comparison with the role played by constitutional lawyers during the referendum campaign is quite revealing, not only in terms of visibility but also in terms of the other two dimensions (partisanship and impact). In fact, their public mobilisation was quite strong considering the topic at stake, which was much closer to their professional interest than most of the political events normally covered by the media. However, the mood of their public interventions suggests that they perceived the reform as a (mostly negative) turning point for the future of the country. Of course, there were different opinions on the reform among constitutional layers too, and some of them did not participate in the referendum campaign. However, we could safely claim that the category was collectively involved in the public debate. Furthermore, the most renowned constitutional lawyers strenuously campaigned for a “no” vote in the media, and this may have affected the final outcome. It probably demonstrates that it is not impossible for public intellectuals and academics to have impact in the public debate, although we do not assume that having impact (Flinders 2013) means pushing the electorate to vote according to the academics’ indications.

Instead, for their part, IPSs only entered the debate shyly and individually, abandoning any notion of playing the game as a professional community in the eyes of the public opinion. In fact, even the few who publicly engaged in the debate resorted to adopting a dramatising narrative of the reform in either direction (automatic drift *vs*. solution to all Italian dysfunctions), and this may have discouraged collective mobilisation, also contributing to IPSs receiving less attention from the media and the public. In this regard, although we assume that many IPSs did actually recognise the importance of being part of this debate, what seems to be emerging is a silent (relative) majority of IPSs who were uneasy with the populist-personalised debate that developed around the reform (Vampa and Vignati 2017). Accordingly, if on the one hand the politically polarised context of the referendum debate may have increased IPSs’ partisanship, on the other hand it was not sufficient for them to shape and affect such debate, just like in Greece and Catalonia crises.

All this may suggest that political scientists’ public engagement is not necessarily linked with specific “crises”, and perhaps not even with the general political focus of the moment. In the Italian case, this is particularly evident if we also take into account, for instance, the composition of the whole comitology that the Italian government organised in response to the first wave of the Sars-Covid-2 pandemic, *the* crisis of our times: various committees were made up of around 450 experts, but political scientists were dramatically absent (Galanti and Saracino 2021). In this regard, our preliminary analysis suggests the importance for future studies in the field to focus more on the extrinsic motivations for, and professional expectations of, engaging in the public debate.

To sum up, we can connect the findings of our press analysis and survey data to the three-dimensional framework developed in this special issue saying that: (a) only a minority of IPSs publicly mobilised in the referendum campaign, with a low overall rate of visibility; (b) among the few who publicly mobilised and/or took part in the media debate in the weeks preceding the referendum, a vast majority took a partisan rather than a neutral stance on the proposed reform, with the “yes” campaigners outnumbering the “no” campaigners. *Partisanship* was thus the prevailing role among the few scholars involved in the debate; (c) however, the majority of Italian voters rejected this position of support for the proposed constitutional reform. This seems to represent a further confirmation of the inconsequential impact of IPSs already demonstrated by our qualitative analysis and the self-perceptions of many scholars.

## Footnotes

1 As it will be more clearly explained in the following sections, we chose the constitutional lawyers as a reference category because they represent the academic-professional group more involved in the public referendum debate, so we assume they could offer useful hints for comparison.

2 The PROSEPS Italian sample was actually based on the 2018 ministerial list, but the differences are negligible.

3 A confirmatory referendum on a constitutional law can be conducted, according to art. 138 of the Italian constitution, if a supermajority of 2/3 of MPs was not reached during the double parliamentary vote on a proposal of constitutional amendment. As is well known, a majority of almost 19.5 million voters (59.1% of the valid votes) rejected the proposal.

4 The first reform—initially promoted by the centre-left but finally approved by the referendum during the II Berlusconi cabinet—amended the Titolo V of the Italian Constitution in order to increase regional powers, strengthening decentralisation. The second possible reform (rejected by the referendum) was proposed and initially approved during Berlusconi II and III cabinet between October 2004 and November 2005. It was supposed to give more power to the Prime Minister, would have ended Italy's symmetric bicameralism and reduced the number of deputies and senators, increasing the power of the regions. Finally, the referendum held in September 2020 approved the reduction in the number of MPs (from 630 to 400 in the Chamber of Deputies and from 315 to 200 in the Senate), strongly wanted by the M5S but (less enthusiastically) supported by the other main Italian parties as well.

5 The PROSEPS survey provides highly representative data regarding the community of political scientists in Italy. Indeed, IPSs showed one of the highest response rates of all participating countries, with more than 60 per cent completing questionnaires (177 out of 290 invitations).

6 The terms “*politologo*” and “*costituzionalista*” have a journalistic nature. This represents the reason why we decided to focus on these two keywords, inflected in their masculine, feminine and plural forms. In fact, we also carried out a countercheck, implementing the same archive search using more “academic” keywords such as “Professor of Political Science/Constitutional Law” and we only had between 0 and 1 occurrences from each period in each newspaper.

7 On the contrary, we did not take into account “*il Sole 24 ore*” in our previous analysis because the organisation of its online archive prevented a keyword search similar to the other newspapers.

8 A more qualitative analysis shows that many interventions/citations referred to foreign political scientists and/or concerned different topics. A possible explanation is perhaps that Italian journalists/columnists feel confident enough about their capacity to comment and interpret internal political issues (each newspaper devoted thousands of articles to the constitutional reform during the 4-month period considered), while they prefer to rely on experts/academics to deal with foreign issues. This is something that deserves to be detected in future studies concerning the relation between academics and the media.

9 The columnist of *la Repubblica* was the only one who has always maintained a neutral stance in all his interventions.

10 Response rates: males 45.6%; females 43.3%. Full professors 44.0%; associate professors 44.2%; researchers with permanent contracts 44.8%; researchers with temporary contracts 53.8%. Northern Universities 43.7%; Central Universities 39.7%; Southern Universities 56.4%. Mega Universities 47.0%; Big Universities 44.4%; Medium Universities 43.6%; Small Universities 35.0%.

11 Motivation: 1 = not at all motivated to engage in the public debate: disagrees that taking part to the public debate is a professional obligation + part of his/her role as social scientist + expands career options; 2 = scarcely motivated: disagrees that taking part to the public debate expands career options, although he/she might agree it is a professional obligation or part of his/her role as social scientist; 3 = quite motivated: agrees that taking part to the public debate is a professional obligation + part of his/her role as social scientist; 4 = extremely motivated: agrees that taking part to the public debate is a professional obligation + part of his/her role as social scientist + expands career options.

12 Social relevance: 1 = extremely pessimistic about PS social relevance in Italy: PS research is scarcely or not at all visible in the public debate + IPSs have no impact compared to other academics; 2 = moderately pessimistic: not at all or scarcely visible + little impact; 3 = moderately optimistic: quite or very visible + little impact; 4 = extremely optimistic: quite or very visible + considerable impact.

13 Media activism: 1 = completely inactive: did not intervene on TV, press or radio over the last 3 years; 2 = scarcely active: sporadic interventions (less than once every three months) on one/two media; 3 = quite active: interventions on two/three media, frequently on at least one; 4 = extremely active: interventions on two/three media, frequently on at least two.
